# Genotype × Environment Shapes Fig Seed Oil Metabolic Fingerprinting

**DOI:** 10.3390/metabo16020127

**Published:** 2026-02-12

**Authors:** Charaf Ed-dine Kassimi, Souhaila Hadday, Souhaila Bouchelta, Ahmed Irchad, Ibtissame Guirrou, Karim Houmanat, Fedoua Diai, Lhoussain Hajji, Lahcen Hssaini

**Affiliations:** 1 Agro-Food Technology and Quality Laboratory, Regional Center of Agricultural Research of Meknes, National Institute of Agricultural Research, Rabat 10090, Morocco; charafeddine.kassimi@inra.ma (C.E.-d.K.); s.hadday@edu.umi.ac.ma (S.H.); ibtissame.guirrou@inra.ma (I.G.); k.houmanat@gmail.com (K.H.); 2 Human Nutrition, Bioactives and Oncogenetics (NHBO), Faculty of Science, University Moulay Ismail, Zitoune, Meknes 50100, Moroccol.hajji@umi.ac.ma (L.H.); 3 National Research Institute for Agriculture, Fisheries and Environment (INRAPE), Ex CEFADER, M’dé, Ngazidja, Moroni P.O. Box 1406, Comoros; irchadahmed04@gmail.com

**Keywords:** fig seed oil, genotype × environment interaction, FTIR-ATR spectroscopy, phenolic compounds, chemometrics

## Abstract

Background/Objectives: Fig (*Ficus carica* L.) seed oil represents an underexplored by-product with considerable nutraceutical potential. However, systematic evaluation of genotype × environment (G × E) interactions affecting its biochemical composition remains limited. This study assessed compositional variability across fig varieties, identified metabolic trade-offs, and developed rapid authentication protocols using FTIR-ATR spectroscopy to support predictive G × E models and marker-assisted selection. Methods: Thirty-seven fig varieties were evaluated across two consecutive harvest years (2023–2024) in Morocco. Conventional biochemical analyses measured total phenolic content (TPC), total flavonoid content (TFC), DPPH and ABTS antioxidant activities, and oil yield. FTIR-ATR spectroscopy characterized spectral variations, with ANOVA assessing effects of year, variety, and G × E interactions. Principal Component Analysis (PCA) discriminated genotypes and years. Results: TPC varied substantially (16.5–115.1 mg GAE/100 g oil), declining 36% from 2023 (48.7 ± 16.6 mg GAE/100 g) to 2024 (31.2 ± 16.6 mg GAE/100 g; F = 1372.84, *p* < 0.001), with TFC showing parallel trends (15.6 vs. 11.8 mg QCE/100 g). DPPH activity increased 34% in 2024 (58.5% vs. 43.7%), while ABTS activity decreased 18.6% from 32.34 ± 14.28% to 26.31 ± 6.10% (*p* < 0.001). Oil yield decreased from 26.7% to 21.2% and negatively correlated with phenolic accumulation (r = −0.49, *p* < 0.001). FTIR-ATR identified diagnostic peaks (e.g., 3012, 2928 cm^−1^), with significant G × E effects (*p* < 0.001). PCA captured 75.4–84.5% variance, discriminating genotypes and years. Stable high-value cultivars included ‘Dottato Perguerolles’, ‘VCR 276/49’, and ‘Ferqouch Jmel’. Conclusions: Genotypic differences and year-to-year environmental conditions significantly influence fig seed oil composition. The observed negative correlation between oil yield and phenolic content indicates a trade-off between lipid biosynthesis and secondary metabolism. FTIR-ATR spectroscopy coupled with multivariate analysis enables reliable variety discrimination and year differentiation, supporting the development of stable cultivars for nutraceutical applications.

## 1. Introduction

*Ficus carica* L., one of the oldest domesticated fruit trees since the Neolithic revolution [1], is a key species in the Mediterranean region, widely recognized for the nutritional richness of its fruits, notably, sugars, fibers, and natural antioxidants [2]. Beyond fresh consumption and traditional processing, fig seeds currently discarded as waste during industrial transformation represent a strategic investment opportunity within the circular economy framework [3,4]. Moreover, various parts of this plant, including leaves, latex, bark, and roots, have traditionally been used for their medicinal properties [5]. Studies have confirmed that figs have functional properties such as anti-diabetes and anti-oxidation [6,7,8,9,10,11,12]. The active ingredients mainly depend on phenols and flavonoids in the fruit, but these studies focus more on the pulp of the fruit, and less on the seeds and their oils. Although FTIR-ATR spectroscopy has been used for variety identification and quality control [13,14,15,16,17], there is no research to verify whether it can accurately capture the changes in the composition of fig seed oil caused by G × E interaction, which makes it difficult to meet the needs of cross-annual quality prediction.

These seeds, characterized by a round shape and yellowish color, vary considerably in number and weight depending on genotype, which suggests potential variability in oil quality [18]. However, no systematic study has been conducted to quantify the synergistic effects of G × E interactions on key quality indicators such as phenols, flavonoid content, antioxidant activity, and oil yield in fig seed oil. In addition, most of the existing studies are single-year single-point experiments, lacking the exploration of the G × E interaction mechanism under inter-annual environmental variation, and cannot provide a basis for variety screening under different climatic conditions.

Furthermore, oil yields from fig seeds fluctuate substantially, reaching up to 40% depending on variety and environmental conditions, reflecting genetic-, geographical-, and pollination-related differences [13,18,19]. These yields are comparable or even superior to those of other fruit seed oils and markedly exceed those from prickly pear seeds [20,21]. For rural cooperatives equipped with basic extraction facilities, this yield potential translates into tangible economic benefits, as seed oil can command premium prices in cosmetic, nutraceutical, and specialty food markets [22,23]. Consequently, this variability highlights an untapped economic potential, especially within the framework of the circular economy and sustainable valorization of local production chains [3,4,19]. Despite this promise, the molecular basis underlying yield variability and its relationship to bioactive compound accumulation remains poorly understood, preventing stakeholders from making informed decisions about cultivar deployment and processing investments.

Biochemically, fig seed oil is distinguished by a high content of essential polyunsaturated fatty acids, primarily α-linolenic acid (C18:3 ω-3) and linoleic acid (C18:2 ω-6), accompanied by notable oleic acid (C18:1 ω-9) levels [19]. These fatty acids contribute significantly to immune function modulation and cardiovascular disease prevention, benefits that are further reinforced by a particularly low omega-6/omega-3 ratio (0.75–0.79) [23,24]. In addition, minor bioactive compounds such as β-sitosterol and γ-tocopherol enhance the antioxidant and nutraceutical properties of the oil [25,26,27].

Although seed oils are predominantly composed of lipophilic compounds (fatty acids, tocopherols, phytosterols), phenolic compounds, despite their hydrophilic nature, can be co-extracted and retained within oil matrices through liquid–liquid extraction using polar solvents such as methanol or methanol–water mixtures [28]. These minor polar compounds, typically present at concentrations ranging from 50 to 940 mg/kg in vegetable oils, contribute significantly to oxidative stability by scavenging free radicals and chelating pro-oxidant metal ions [29]. Beyond lipid protection, they demonstrate biological activities including protective effects against LDL oxidation and cardiovascular diseases [30]. Phenolic compounds extracted following an adapted method [31] were quantified by the Folin–Ciocalteu colorimetric assay [32], revealing a total phenolic content of 79.5 mg gallic acid equivalent per 100 g oil [25].

However, oil chemical composition appears markedly influenced by both varietal and environmental factors, with recent studies reporting significant biochemical variability among Moroccan cultivars and other genotypes [13,18]. Critical unresolved questions remain: whether these nutritional attributes remain stable across harvest years or exhibit genotype-dependent plasticity under variable climatic regimes, whether phenolic accumulation and lipid biosynthesis operate independently or exhibit metabolic trade-offs that could constrain simultaneous optimization of both traits, and whether Fourier-transform infrared spectroscopy (FTIR-ATR) signatures can capture genotype × environment-driven compositional shifts with sufficient resolution to enable rapid authentication and quality prediction across multiple seasons [13,14,15,16,17].

We hypothesize the following: (1) Genotypic differences and cross-annual environmental variations jointly drive the changes in the biochemical characteristics of fig seed oil, and there is a metabolic trade-off between secondary metabolites such as phenols and flavonoids and oil synthesis. (2) The composition change caused by G × E interaction can be captured by the characteristic peaks of FTIR-ATR spectra. (3) The combination of biochemical analysis and spectral technology can realize variety identification and cross-year quality prediction, and improve the clarity.

In this context, the present study aims to investigate, over two consecutive years (2023 and 2024), the biochemical and spectral variability (FTIR-ATR) of oils obtained from seeds of 37 *Ficus carica* L. cultivars. The integrated analysis of total polyphenols, flavonoids, DPPH, ABTS antioxidant assays, and FTIR spectra is expected to offer a rapid and reliable approach for characterization, varietal discrimination, and provide technical support for directional screening of fig seed oil varieties, cross-year quality prediction, and stable application in food and cosmetics.

## 2. Materials and Methods

### 2.1. Plant Material and Experimental Design

A diverse collection of thirty-seven fig tree genotypes (*Ficus carica* L.) was selected from an ex situ genetic resource maintained at the experimental station of the National Institute of Agricultural Research (INRA) in Meknes, Morocco. This collection includes twenty-three local clones and fourteen exotic cultivars (Table 1).

**Figure 1:**
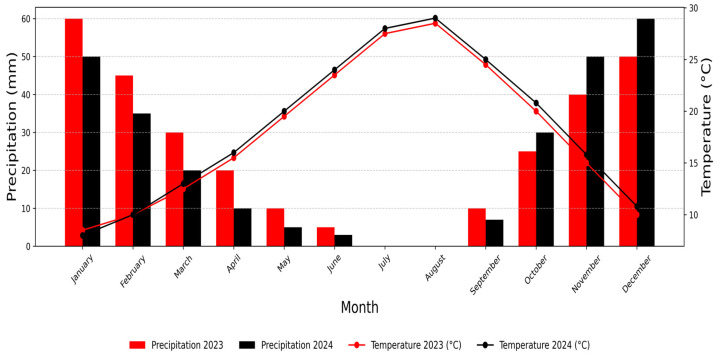
Comparison of monthly precipitation (mm) and temperature (°C) between 2023 and 2024.

The trees, aged eighteen years and pruned into a cup shape, were arranged in a randomized complete block design with three individual trees per genotype spaced 5 m by 3 m apart. The orchard is maintained under rainfed conditions with supplementary drip irrigation during critical dry periods, following standard cultural practices including annual winter pruning and integrated pest management. Phenotypic evaluations were carried out at full maturity during critical periods from July to September in 2023 and 2024. Climatic data for both growing seasons were recorded using an on-site weather station. The 2023 season was characterized by moderate thermal conditions, with a mean temperature of 17.9 °C and a cumulative rainfall of 295 mm during the reproductive period (Figure 1). In contrast, the 2024 season exhibited slightly higher thermal stress (mean 18.3 °C) and lower precipitation (270 mm), consistent with regional climate variability patterns.

### 2.2. Fruit Sampling

Figs were collected at full ripeness during the summer season. Genotypes were selected based on fruit size and seed count. Full maturity was identified when approximately three-quarters of the fruit surface exhibited a reddish-purple hue, and the fruit could be easily detached from the branch with gentle hand pressure, following established maturity indices for *Ficus carica* L. [19]. To obtain a representative sample, approximately 15–20 fruits were randomly picked from different sections of the canopy at a uniform height of 160 cm from the three replicate trees per genotype. Fruits were immediately transported to the laboratory in ventilated containers and processed within 24 h of harvest to minimize post-harvest biochemical changes.

### 2.3. Seed and Oil Extraction

Seeds were manually separated from the fig pulp using a 10% ethanol solution to prevent microbial growth and enzymatic degradation. The mixture was agitated for about 10 min using a magnetic stirrer at 200 rpm and then allowed to rest, enabling seeds to rise to the surface. The floated yellow, spherical seeds were collected, rinsed thoroughly with distilled water until the rinse water ran clear (typically 3–4 rinses), and air-dried at ambient temperature (22–25 °C) for 24 h on absorbent paper with periodic turning to ensure uniform drying. The dried seeds were subsequently ground to a fine powder using an IKA A11 basic grinder (IKA-Werke GmbH & Co. KG, Staufen, Germany) operating at 20,000 rpm for 30 s, achieving a particle size <0.5 mm. Oil was extracted using a Soxhlet extraction technique. A total of 20 g of finely ground seed samples were placed in cellulose thimbles and extracted with 150 mL of n-hexane for 4 h. After extraction, the solvent was removed under reduced pressure using a Büchi Rotavapor R-200 at 40 °C. The oil yield was then determined in accordance with the procedure described by Kassimi et al. (2025) [13].

Oil yield (%) = [(M1 − M0)/M2] × 100(1)

where

M0 represents the weight of the empty flask in grams (g);
M1 corresponds to the weight of the flask following solvent evaporation (g);
M2 denotes the weight of the seed powder (g).

All extractions were performed in triplicate, and oil yield is reported as mean ± standard deviation. The average weight of the residual oil cake was also determined. Extracted oils were stored in dark glass containers fitted with Teflon-lined caps, flushed with nitrogen gas to prevent oxidation, at 4 °C, and analyzed within two weeks of extraction until further analysis. Distilled water was further purified using a Milli-Q system (Millipore, Bedford, MA, USA) to achieve ultrapure water (18.2 MΩ·cm resistivity) for all analytical procedures.

### 2.4. Seed Oil Analysis

#### Chemical Inputs

All reagents employed in this study were of analytical grade and sourced from Sigma-Aldrich (Saint Louis, MO, USA).

### 2.5. Biochemical Analysis

#### 2.5.1. Phenol Extraction

To determine the total phenolic content, total flavonoid content, and antioxidant capacity, the extraction of compounds followed the method outlined by Tsimidou et al. (1992), with slight modifications [31]. In brief, 2 g of oil were dissolved in 10 mL of hexane, to which 4 mL of 60% methanol (*v*/*v*) in ultrapure water was added. The mixture was gently agitated on an orbital shaker (150 rpm) at room temperature (22–25 °C) for 2 h, protected from light using aluminum foil to prevent degradation of light-sensitive compounds. After extraction, the upper methanolic phase was separated using a separatory funnel and filtered through Whatman No. 1 filter paper (Whatman International, Brentford, UK). This extraction was repeated three times to ensure maximum recovery and reproducibility. The collected filtrates were pooled, washed with an additional 10 mL of hexane to eliminate remaining lipid residues, and stored in amber vials at 4 °C for no longer than 48 h until analysis.

#### 2.5.2. Total Phenolic Content (TPC)

Total phenolic content (TPC) was determined using a modified version of the Folin–Ciocalteu assay, based on the method described by Singleton et al. (1999) [33]. In this procedure, 50 μL of the extract was mixed with 3 mL of ultrapurewater, followed by the addition of 250 μL of Folin–Ciocalteu reagent and 750 μL of a 7% *w*/*v* sodium carbonate solution. The mixture was gently mixed by inversion at room temperature for 8 min. Then, 950 μL of ultrapure water was added, and the reaction was left to develop in the dark for 2 h at room temperature. Absorbance was measured at 765 nm using a Jasco V-530 UV-Vis spectrophotometer (Hachioji, Tokyo, Japan), with a blank containing all reagents except the sample extract for baseline correction. Gallic acid at concentrations ranging from 10 to 100 μg/mL served as the standard to generate the calibration curve (R^2^ > 0.987), and TPC results were expressed as milligrams of gallic acid equivalents (GAE) per 100 g of oil. All measurements were performed in triplicate, and results are reported as mean ± standard deviation.

#### 2.5.3. Total Flavonoid Content (TFC)

The total flavonoid content (TFC) was assessed following the protocol established by Favati et al. (1994) [32]. In short, 0.5 mL of the extract was combined with 0.5 mL of a 2% *w*/*v* aluminum chloride solution prepared in methanol. The mixture was left to react at room temperature for 15 min in the dark, after which the absorbance was measured at 430 nm, using methanol as the blank reference. Quercetin at concentrations of 5–50 μg/mL was used as the standard (R^2^ > 0.998), and TFC was expressed as milligrams of Quercetin equivalents (QCE) per 100 g of oil. All measurements were performed in triplicate.

#### 2.5.4. Free Radical Scavenging Activity (DPPH Assay)

Free radical scavenging activity (FRSA) was measured using the DPPH (2,2-diphenyl-1-picrylhydrazyl) assay, adapted from the method of Brand-Williams et al. (1995) [34]. In this procedure, 50 μL of the oil extract was mixed with 950 μL of a methanolic DPPH solution (0.030 mg/mL, freshly prepared before each assay). The mixture was incubated in the dark for 60 min at room temperature to avoid light-induced degradation. Following incubation, absorbance was recorded at 515 nm using a spectrophotometer, with a blank prepared by substituting the sample with methanol instead of ultrapure water. The DPPH radical scavenging activity was calculated as a percentage inhibition using the following formula:

DPPH inhibition (%) = [(Ac − Ae)/Ac] × 100

where Ac is the absorbance of the control, and Ae is the absorbance of the sample. All measurements were performed in triplicate.

#### 2.5.5. Radical Cation Decolorization (ABTS Assay)

Antioxidant capacity was assessed using the ABTS (2,2′-azino-bis (3-ethylbenzothiazoline-6-sulfonic acid) radical cation decolorization assay, based on the method of Re et al. (1999) [35] with slight modifications. The ABTS•+stock solution was prepared by reacting 7 mM ABTS with 2.45 mM potassium persulfate in ultrapure water and storing it in the dark at room temperature for 12–16 h before use. The working solution was obtained by diluting the stock with methanol to an absorbance of 0.70 ± 0.02 at 734 nm. In this test, 10 μL of the oil extract was mixed with 990 μL of ABTS^+^ solution, which was generated by reacting ABTS with potassium persulfate. The mixture was incubated in the dark at room temperature for 6 min. Absorbance was then measured at 734 nm, using methanol as the blank. The percentage of radical scavenging activity was calculated using the following formula:

ABTS inhibition (%) = [(Ac − Ae)/Ac] × 100,

where Ac and Ae represent the absorbance of the control and the sample, respectively.

### 2.6. Variety Coding for Statistical Analysis

For multivariate statistical purposes, each of the thirty-seven fig (*Ficus carica* L.) varieties was assigned a numerical code to simplify data visualization and interpretation in the statistical plots (Table 2). This numerical identification was consistently applied throughout the biochemical and the FTIR-ATR statistical analyses. This coding approach allowed for a clear graphical representation in the PCA score plots (Figures 4 and 9) without overcrowding the visualization with full cultivar names.

### 2.7. FTIR-ATR Spectroscopy

Fourier transform infrared (FTIR) spectra of fig seed oil were obtained using a Bruker spectrometer (Bruker Optics Inc., Ettlingen, Germany), scanning the wavenumber range from 4000 to 450 cm^−1^ with a resolution of 4 cm^−1^. Analyses were conducted at room temperature with approximately 50 μL of oil applied per measurement to ensure complete coverage of the crystal surface. Each spectrum represents the average of multiple replicates, with 128 scans recorded for each sample to improve signal-to-noise ratio. Prior to sample measurement, a background spectrum was collected using a clean germanium crystal in air and subsequently subtracted from the sample spectra automatically.

To maintain measurement accuracy, the ATR crystal was thoroughly cleaned with analytical-grade ethanol and warm ultrapure water between samples, then dried with absorbent paper. Baseline correction was applied to all spectra to minimize potential interference from atmospheric CO_2_ and water vapor. ATR correction was carried out with parameters set at a 45° angle of incidence, single-bounce ATR reflection, and a sample refractive index of 1.5, typical for vegetable oils. The maximum penetration depth was fixed at 50 μm over a 1.8 mm crystal surface [36].

In ATR spectroscopy, the IR light reflects internally within the high refractive index crystal, generating an evanescent wave that interacts with the sample’s lower refractive index surface. to a depth of approximately 0.5–2 μm, providing information about the molecular composition at the oil–crystal interface. Spectral intensities corresponding to the molecular fingerprints were quantified using Essential FTIR software (version 3.00, Operant LLC, Burke, VA, USA) [37]. Integrated band areas were calculated for five characteristic peaks: 3012 cm^−1^ (=C–H stretching of unsaturated fatty acids), 2928 cm^−1^ (–CH_2_ asymmetric stretching), 1745 cm^−1^ (C=O ester stretching of triglycerides), 1375 cm^−1^ (CH_3_ symmetric bending), and 1050 cm^−1^ (C–O stretching of primary alcohols and esters). Baseline correction was performed using a linear baseline drawn between the integration limits.

### 2.8. Data Analysis

All data analyses were performed using Python (v3.10), with pandas (v1.5.3), numpy (v1.24.2), matplotlib (v3.7.1), seaborn (v0.12.2), scipy (v1.10.1), and scikit-learn (v1.2.2) libraries being employed for data processing and visualization. Statistical significance was set at α = 0.05 for all tests unless otherwise specified.

#### 2.8.1. Biochemical Data Analysis

Descriptive statistics were first calculated for all biochemical parameters, including total phenolic content (TPC), total flavonoid content (TFC), antioxidant capacities (DPPH and ABTS), oil yield, and seed yield.

The main design factors, including the year and the variety, were assessed for their effect on the abovementioned parameters individually and in the combination of both (Year × Variety), through a two-way Analysis of Variance (ANOVA) using the Type III sum of squares method to account for unbalanced designs. Normality of residuals was assessed using the Shapiro–Wilk test, and homogeneity of variances was verified using Levene’s test. When significant effects were detected (*p* < 0.05), pairwise comparisons were further examined using Tukey’s HSD test honest significant difference test with Bonferroni correction for multiple comparisons.

Data distribution patterns were visualized using boxplots, and relationships among biochemical traits were explored through a correlation heatmap based on Pearson’s correlation coefficients. A PCA biplot was also constructed to identify global trends and similarities among varieties across years. PCA was performed on standardized data (z-score normalization) to give equal weight to all variables regardless of their measurement units. The number of principal components retained was determined based on the Kaiser criterion (eigenvalues > 1) and cumulative explained variance exceeding 70%.

#### 2.8.2. FTIR Integrated Area Analysis

For the FTIR analysis, the integrated absorbance areas of the main spectral peaks (3012, 2928, 1745, 1375 and 1050 cm^−1^) were computed directly from the raw spectra without preprocessing using trapezoidal numerical integration after linear baseline correction.

A two-way ANOVA was then applied to each integrated area to examine the effects of year, variety and their interaction following the same statistical assumptions and procedures described in Section 2.8.1. Boxplots were generated to compare the distribution of integrated absorbance values between years, while PCA biplots were used to visualize the overall grouping and variance structure among samples based on FTIR peaks. PCA on FTIR data was performed on mean-centered integrated areas without additional scaling to preserve the relative magnitudes of spectral features.

## 3. Results

### 3.1. Seed Yield per Fruit and Oil Yield

The seed yield per fruit and oil yield exhibited substantial variation among the thirty-seven fig genotypes analyzed over the two consecutive seasons (2023 and 2024). Both traits showed a strong dependence on the genetic background and a noticeable influence of the growing year, suggesting that genotype × environment interactions play a key role in determining the productivity and oil accumulation potential of fig seeds.

In 2023, seed yield varied from 1.023 ± 0.019 g for ‘Jeblia’ to 3.608 ± 0.1 g for ‘Bourjassate Noire’, with an average of 2.00 g (Table 3). In 2024, values ranged from 0.387 ± 0.055 g ‘INRA 1304’ to 5.053 ± 0.07 g ‘Bourjassate Noire’, averaging 2.25 ± 1.12 g. Genotypes ‘VCR 276/49’ and ‘Bourjassate Noire’ maintained high yields across both seasons.

Oil content showed substantial variability between genotypes and seasons. In 2023, it ranged from 10.55 ± 0.75% for ‘Jeblia’ to 35.56 ± 1.16% for ‘Sucre Vert’, averaging 25.6 ± 0.05%. Genotypes ‘Sucre Vert’, ‘Hamra’, ‘White Adriatic’, and ‘INRA 2304’ exceeded 33%.

In 2024, a marked reduction was observed, with values from 6.31 ± 0.69% (‘INRA 1304’) to 37.82 ± 0.43% ‘Ferqouch Jmel’, representing an overall 20.5% drop (26.6% vs. 21.2%, *p* < 0.001). Nonetheless, ‘Ferqouch Jmel’ and ‘Noukali’ sustained high yields.

### 3.2. Biochemical Characterization and Statistical Evaluation

#### Descriptive Analysis of Biochemical Parameters

- Yearly trends in biochemical composition and yield traits

**Figure 2:**
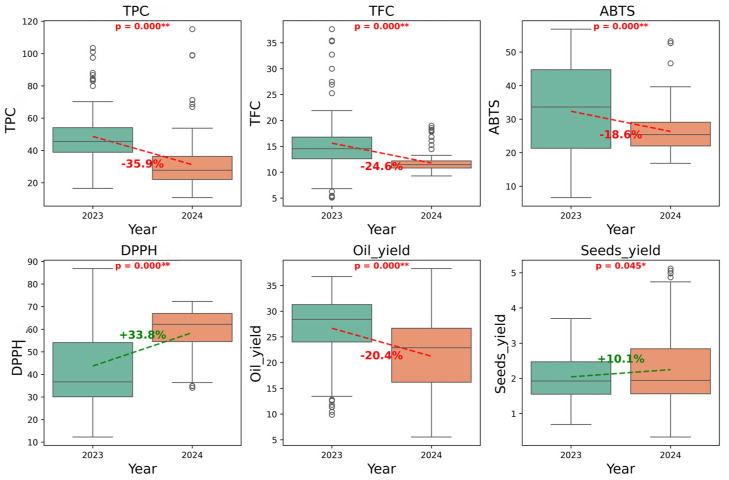
Yearly variation in biochemical and agronomic parameters of fig seed oil samples (2023 vs. 2024). Boxplots illustrate the distribution of biochemical and agronomic parameters, with boxes representing the interquartile range and the median indicated by the central line. Circles represent individual data points. Dashed lines indicate relative increases (green) or decreases (red) and *p*-values from paired *t*-tests (* *p* ≤ 0.05; ** *p* ≤ 0.01).

The biochemical profiling of the thirty-seven fig genotypes over the 2023 and 2024 seasons revealed substantial interannual and genotypic variability (Table 4; Figure 2).

The average total phenolic content (TPC) significantly decreased from 48.68 ± 16.59 mg GAE/100 g oil in 2023 to 31.21 ± 16.64 mg GAE/100 g oil in 2024, representing a 36% reduction (*p* < 0.001). The wider dispersion observed in 2023 indicates greater genetic variability in phenolic responses. Flavonoid content dropped from 15.62 ± 5.86 mg QCE/100 g oil in 2023 to 11.78 ± 1.88 mg QCE/100 g oil in 2024. The reduced variability in 2024 suggests a more uniform metabolic response across genotypes. DPPH scavenging activity increased from 43.70 ± 20.94% in 2023 to 58.49 ± 10.78% in 2024, a significant 34% improvement (*p* < 0.001), while ABTS activity decreased from 32.34 ± 14.28% to 26.31 ± 6.10%, a 18.6% reduction (*p* < 0.001). Oil content decreased from 26.68 ± 6.77% in 2023 to 21.25 ± 7.42% in 2024, while seed yield per fruit slightly increased from 2.04 ± 0.66 g to 2.25 ± 1.12 g.

- Varietal trends in biochemical composition and yield traits

**Figure 3:**
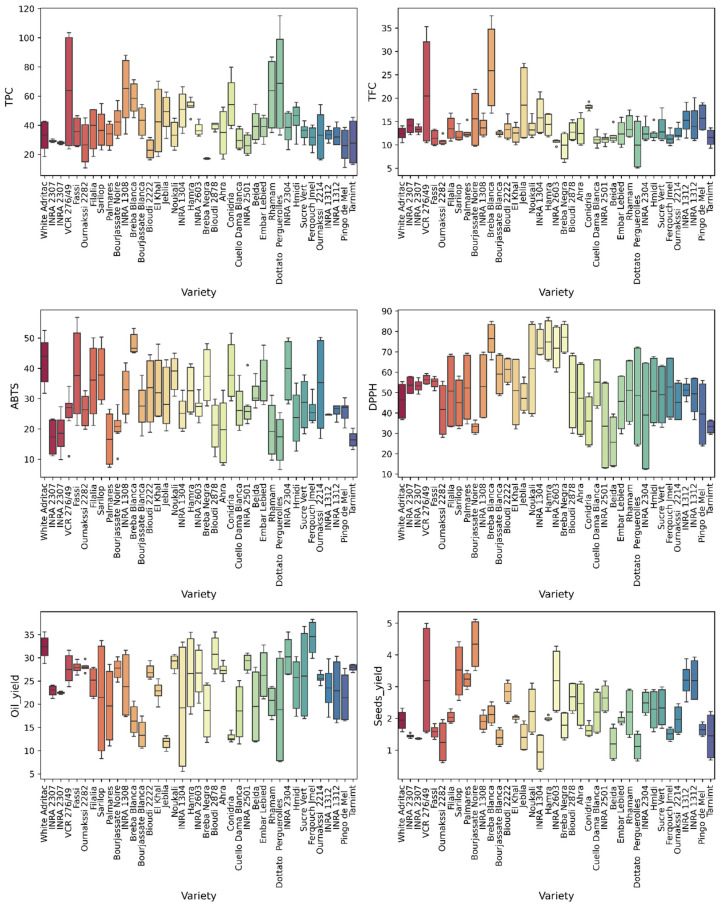
Distribution of biochemical and agronomic parameters across fig varieties, revealing genotype-driven differences. Boxes representing the interquartile range and the median indicated by the central line. Circles represent individual data points. Each variety is represented by a different color.

Marked differences were observed between genotypes (Figure 3). ‘Dottato Perguerolles’, ‘VCR 276/49’, ‘Rhamam’, and ‘INRA 1304’ exhibited the highest TPC (>70 mg GAE/100 g). ‘Breba Negra’, ‘Bioudi 2222’, and ‘INRA 2307’ showed lower TPC. For TFC, ‘Breba Blanca’, ‘VCR 276/49’, and ‘Jeblia’ maintained high levels. ‘Breba Blanca’, ‘Breba Negra’, and ‘Hamra’ displayed strong DPPH activity, while ‘Breba Blanca’ and ‘White Adriatic’ showed marked ABTS responses. ‘Ferqouch Jmel’ and ‘White Adriatic’ presented the highest oil contents (>30%), while ‘Jeblia’ and ‘Conidria’ showed lower yields. ‘Bourjassate Noire’, ‘Sarilop’, and ‘VCR 276/49’ displayed the highest median seed weights.

### 3.3. Multivariate and Univariate Statistical Analyses

ANOVA revealed highly significant effects (*p* < 0.001) of Year, Genotype, and their interaction on all parameters (Table 5). The Year effect was particularly pronounced for DPPH (F = 6199.06) and oil content (F = 1864.59). The significant varietal effect demonstrates substantial genetic diversity, although F-values (35.9–550.8) were lower than year effects. The Year × Genotype interaction (F = 28.6–367.3) reveals pronounced variations in genotype ranking between years.

**Figure 4:**
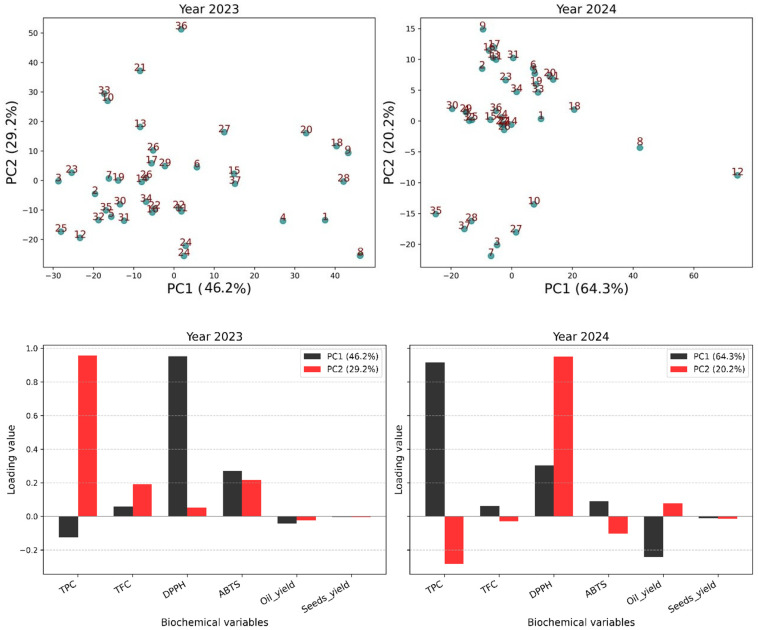
PCA illustrating the relationships between biochemical and yield parameters across 37 fig varieties for the 2023 and 2024 harvest years. Points in the score plot correspond to sample numerical codes listed in Table 2.

The PCA revealed clear differentiation patterns (Figure 4). In 2023, PC1 and PC2 explained 75.4% of the variance (46.2% and 29.2%), while in 2024, this reached 84.5% (64.3% and 20.2%).

In 2023, PC1 was associated with antioxidant activity (DPPH, ABTS), while PC2 was dominated by TPC. In 2024, TPC became the main contributor to PC1, with oil content negatively correlated, and PC2 was explained by DPPH.

Score plots illustrated these relationships. In 2023, dispersion was wide with distinct clusters: ‘Conidria’ (9), ‘Hmidi’ (18), ‘INRA 1308’ (20), and ‘Noukali’ (28) occupied the upper-right quadrant, while ‘Jeblia’ (27), ‘Ferqouch Jmel’ (15), and ‘INRA 2501’ (24) clustered toward the lower quadrants. In 2024, the distribution was more compact.

**Figure 5:**
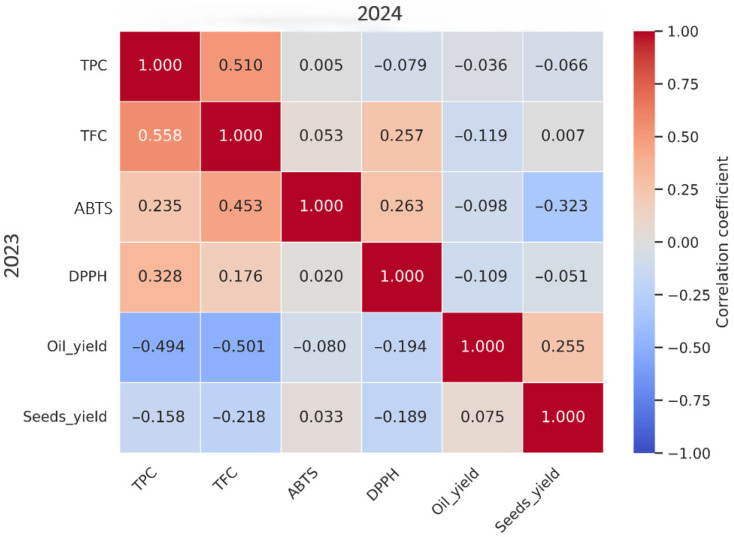
Combined heatmap of correlations between biochemical and yield traits for the 2023 and 2024 seasons. Correlations for the 2024 season are shown in the upper triangle, while correlations for the 2023 season are in the lower triangle.

The heatmap (Figure 5) revealed moderate positive associations between TPC and TFC (r = 0.56 in 2023; r = 0.51 in 2024). DPPH showed positive correlation with TPC (r = 0.33 in 2023) and TFC (r = 0.18–0.26), while ABTS exhibited weak associations (r < 0.3).

Oil content showed a negative correlation with TPC (r = −0.49 in 2023) and TFC (r = −0.50 in 2023). Seed yield was weakly correlated with oil content (r = 0.25 in 2023).

### 3.4. Characteristics of FTIR-ATR Signatures

**Figure 6:**
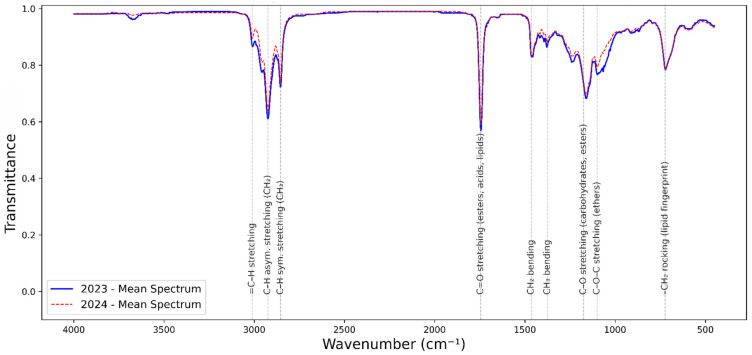
Average FTIR-ATR spectra of fig oils from 37 genotypes across two years, 2023 and 2024, showing characteristic absorption bands. Spectra represent mean absorbance values (n = 37 per year). Major functional group peaks are highlighted and labeled according to Table 6.

The averaged FTIR spectra of the thirty-seven fig genotypes recorded in 2023 and 2024 exhibited highly comparable global profiles (Figure 6).

C–H stretching vibrations of aliphatic chains were identified by sharp peaks around 2928 cm^−1^ and 2855 cm^−1^. A weaker band near 3012 cm^−1^ was attributed to =C–H stretching of olefinic groups. The intensity ratio between the 2928 and 2855 cm^−1^ peaks (typically 1.1–1.3) provides information on chain length distribution.

The strong absorption at 1745 cm^−1^ was assigned to the C=O ester stretching vibration of triglycerides. Other key peaks include 1500 and 1375 cm^−1^, associated with C–H bending, aromatic ring vibrations from phenolics, and methyl bending of lipids. The 1175 cm^−1^ band was attributed to C–O stretching vibrations of carbohydrates, while the 723 cm^−1^ band was related to –CH_2_ rocking.

In 2024, the spectra tended to display higher absorbance intensities in the C=O ester band (~1745 cm^−1^) compared to 2023.

**Table 6:** FTIR peaks assignments for functional groups found in fig seed oil (*Ficus carica* L.).

Wavenumber (cm^−1^)	Functional Group	Assignment	References
3012	=C–H stretching	Olefinic groups, unsaturated fatty acids	[38]
2928	–CH_2_ stretching	Aliphatic chains	[39,40]
2855	–CH_3_ stretching	Terminal groups of fatty acids	[39,40]
1745	C=O ester stretching	Triglycerides, lipids	[41,42]
1500	C–H bending/aromatic ring vibrations	Phenolics, residual proteins	[40,41,42,43]
1375	CH_3_ bending	Lipids	[43]
1175	C–O stretching	Carbohydrates, esterified sugars	[40,41,42,43,44]
1050	C–O stretching	Polysaccharides	[40,41,42,43,44]
723	–CH_2_ rocking	lipid fingerprint	[45,46]

### 3.5. Descriptive Analysis of Integrated Areas

#### 3.5.1. Yearly Differentiation of Integrated Areas

**Figure 7:**
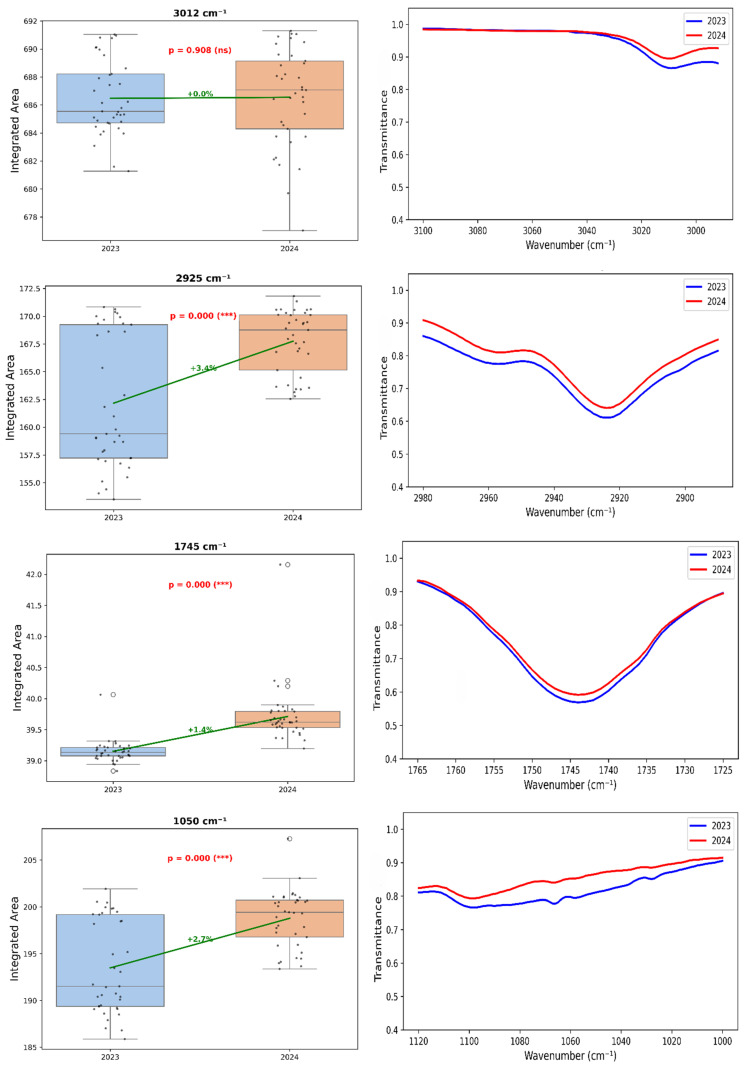

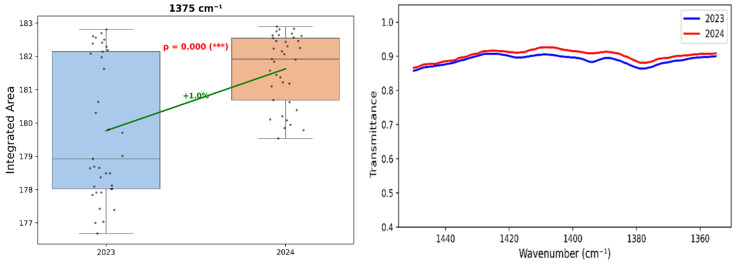
Overall effect of crop year (2023 vs. 2024) on main FTIR bands. (**Left**) boxplots of integrated band areas, boxes representing the interquartile range and the median indicated by the central line. Circles represent individual data points. Solid lines indicate relative increases (green) or decreases (red) and *p*-values from paired *t*-tests (*** *p* ≤ 0.001; ns = not significant). (**Right**) mean FTIR spectra per year (2023 blue, 2024 red) showing spectral peaks used for integration.

FTIR-ATR absorption area evaluation revealed subtle but significant interannual differences (Figure 7). The =C-H elongation at 3012 cm^−1^ showed a slight shift in 2024. Median intensity at 2928 cm^−1^ increased in 2024. The carbonyl peak at 1745 cm^−1^ displayed higher and less variable areas in 2024, with a 2–5% area increase. The bands at 1375 cm^−1^ and 1050 cm^−1^ showed higher areas in 2024.

#### 3.5.2. Varietal Differentiation of Integrated Areas

**Figure 8:**
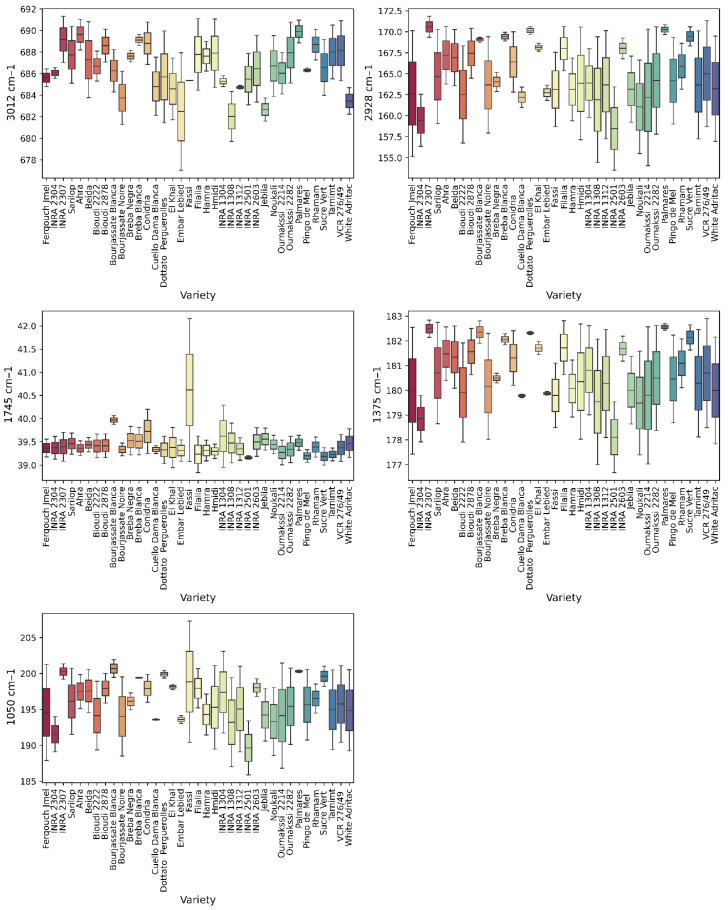
Distribution of integrated FTIR–ATR band areas across fig varieties, revealing genotype-driven differences. Each variety is represented by a different color.

Varietal boxplots (Figure 8) reveal substantial heterogeneity. At 3012 cm^−1^, ‘Palmares’, ‘Ahra’, and ‘Breba Blanca’ exhibited the highest intensities, while ‘INRA 1304’, ‘Jeblia’, and ‘Embar Lebied’ showed the weakest responses. At 2928 cm^−1^, ‘Palmares’, ‘INRA 2307’, and ‘Dottato Perguerolles’ displayed the highest median values. At 1745 cm^−1^, ‘Dottato Perguerolles’ and ‘Bourjassate Blanca’ exhibited the most marked signals. The bands at 1375 and 1050 cm^−1^ revealed genotypic clustering, with ‘Palmares’, ‘INRA 2307’, and ‘Bourjassate Blanca’ showing high median intensities.

### 3.6. Multivariate Analysis of FTIR-ATR Integrated Areas

The multivariate analysis revealed significant sources of spectral variation (Table 7). All five characteristic absorption bands showed highly significant (*p* < 0.001) effects of year, variety, and their interaction. Interannual variability exerted a pronounced influence, particularly within the aliphatic –CH_2_ stretching band (2928 cm^−1^; F = 73,421.83) and the carbohydrate-associated band (1050 cm^−1^; F = 9435.603).

The PCA (Figure 4) provided an overview of biochemical differentiation. For 2023, PC1 and PC2 explained 77.6% and 22.3% of the variance, respectively, while in 2024, PC1 and PC2 accounted for 69.3% and 29.2%.

In 2023, PC1 was characterized by strong positive loadings for lipid, protein, and carbohydrate bands (3012, 2928, 1375, 1050 cm^−1^), while PC2 showed a marked positive contribution from the carbonyl band (1745 cm^−1^). In 2024, all spectral regions exhibited positive contributions on PC1, although the carbonyl band retained the weakest contribution. On PC2, the carbonyl band showed the highest positive contribution.

**Figure 9:**
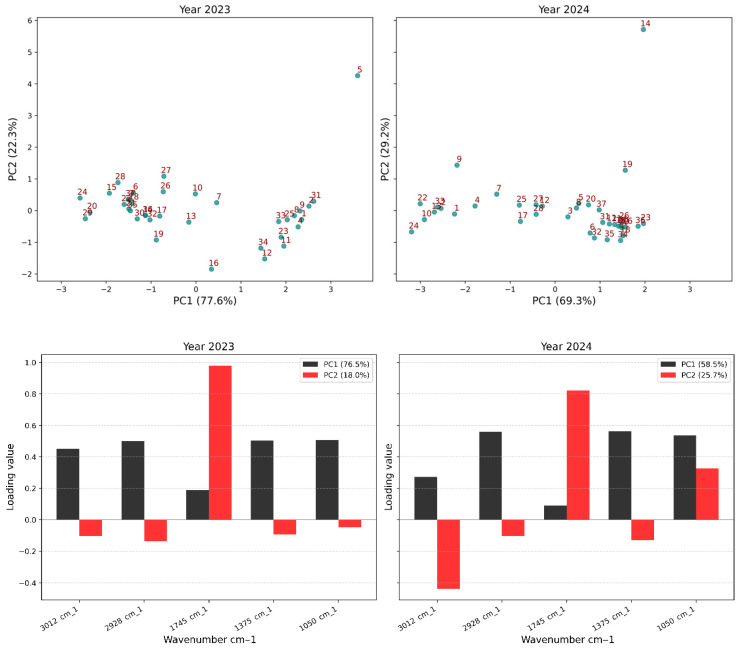
Multivariate differentiation of fig oils from 37 varieties based on FTIR spectral areas for 2023 and 2024 as revealed by PCA plots. Points in the score plot correspond to sample numerical codes listed in Table 2.

Score plots (Figure 9) revealed clear varietal groupings. In 2023, three groups were distinguished: Group I (‘INRA 2501’, ‘Ferqouch Jmel’, ‘Noukali’, ‘Sarilop’) with weaker lipid contributions; Group II (‘Breba Negra’, ‘INRA 1304’, ‘Cuello Dama Blanca’) with intermediate profiles; Group III (‘El Khal’, ‘INRA 2307’, ‘Palmares’, ‘Sucre Vert’, ‘Bioudi 2878’) with higher lipid and carbohydrate absorbances. ‘Bourjassate Blanca’ was isolated. In 2024, distribution was more compact, but three subgroups remained discernible.

## 4. Discussion

### 4.1. Seed Yield t and Oil Yield

This variability reflects significant genetic differences in seed development, consistent with prior observations on *Ficus carica* L. [13,19]. The 9% reduction in precipitation (270 vs. 295 mm) and temperature increase (+0.4 °C) induced water stress that limited assimilate allocation to seeds in sensitive genotypes [47]. Genotypes ‘VCR 276/49’ and ‘Bourjassate Noire’ maintained high yields across both seasons, confirming their genetic stability.

Oil content results were in line with previous data [19,48]. Genotypes exceeding 33% indicated strong predisposition to lipid biosynthesis, comparable to established oil seeds [23].

The overall 20.5% drop in 2024 reflects water stress that restricted photosynthate supply and redirected carbon allocation toward protective compounds rather than storage lipids [49,50]. The resilience of ‘Ferqouch Jmel’ and ‘Noukali’ highlights their potential for production in drought-prone Mediterranean areas.

### 4.2. Biochemical Characterization: Year and Varietal Effects

- Yearly effects in biochemical composition and yield traits

The higher phenolic accumulation in 2023 likely reflects stronger activation of secondary metabolism under more favorable or moderately stressful environmental conditions, as temperature and water stress are known to stimulate phenol biosynthesis [2,51]. However, the relationship between stress and phenolic accumulation is complex and non-linear. Moderate stress generally enhances secondary metabolism through upregulation of the phenylpropanoid pathway, while severe stress can suppress biosynthetic capacity due to limited photosynthate availability [52,53]. The 2023 season, with its more balanced temperature and humidity regime, likely provided optimal conditions for phenol biosynthesis without imposing severe metabolic limitations. The wider dispersion observed in 2023 also indicates greater genetic variability in phenolic responses, suggesting that environmental conditions that year allowed fuller expression of genetic differences in secondary metabolism.

The reduced variability in 2024 suggests a more uniform metabolic response across genotypes, indicating environmental constraints that limited phenotypic expression of genetic variability. Flavonoid synthesis is tightly regulated by light exposure, UV intensity, and temperature [54,55], which may explain interannual variations. Greater solar radiation and reduced cloud cover, typically associated with drier seasons (2024), should theoretically favor flavonoid synthesis as a UV-protective response. However, the observed decrease suggests that water deficit was the predominant limiting factor, restricting carbon allocation to secondary metabolism despite potentially favorable light conditions [56].

These divergent responses between DPPH and ABTS suggest that antioxidant potential depends on compound type, with DPPH and ABTS reacting differently to hydrophilic versus lipophilic antioxidants [57,58]. The more lipophilic DPPH radical preferentially reacts with phenolic compounds soluble in organic solvents and tocopherols, while the water-soluble ABTS cation radical primarily measures hydrophilic antioxidants like phenolic acids and glycosylated flavonoids [59]. The increased DPPH activity in 2024, despite lower total phenol content, suggests a qualitative shift toward more lipophilic or structurally modified phenolic compounds with higher radical scavenging efficiency per molecule. This could reflect stress-induced structural modifications of phenols, such as increased methylation or reduced glycosylation, enhancing lipid solubility and antioxidant potency [60].

Despite the apparent increase in average seed yield, the high standard deviation and greater dispersion in 2024 indicate substantial variability, with some genotypes showing drastic reductions while others increased. This inverse relationship implies that while fruit biomass was preserved, lipid biosynthesis was less efficient under 2024 climatic conditions. Comparable observations have been reported in other oilseed species under environmental stress [49,50]. The maintenance or slight increase in seed weight coupled with reduced oil content suggests that carbon was preferentially allocated to structural carbohydrates and proteins rather than storage lipids under drought conditions, consistent with metabolic adjustments in seed filling processes where carbohydrate synthesis and protein accumulation are prioritized over lipid biosynthesis during water limitation [61].

Overall, descriptive statistics highlight strong environmental modulation of fig seed phenolic and lipid metabolism. The 2023 season favored phenol and flavonoid accumulation, while 2024 enhanced antioxidant reactivity (DPPH), a pattern often linked to compensatory metabolic adjustments in response to stress [62]. These results support our hypothesis that interannual environmental conditions induce coordinated biochemical adjustments involving metabolic trade-offs between secondary metabolism and primary lipid biosynthesis.

- Varietal effects in biochemical composition and yield traits

High TPC in ‘Dottato Perguerolles’, ‘VCR 276/49’, ‘Rhamam’, and ‘INRA 1304’ confers enhanced oxidative stability [26]. Lower TPC in ‘Breba Negra’, ‘Bioudi 2222’, and ‘INRA 2307’ suggests a trade-off toward lipid metabolism.

Antioxidant assays highlighting unequal contributions to hydrophilic and lipophilic systems confirm findings by Prior et al. (2005) [57].

The negative relationship between phenolic synthesis and lipid biosynthesis in ‘Ferqouch Jmel’, ‘White Adriatic’, ‘Jeblia’, and ‘Conidria’ illustrates metabolic competition [63]. This metabolic competition implies that selection for maximum oil yield may reduce phenolic content and vice versa [64].

Differences in seed yield reflect variations in reproductive allocation that influence oil extraction potential [64].

### 4.3. Interpretation of Multivariate and Univariate Analyses of Biochemical Parameters

The high F-values confirm substantial genetic and environmental influences. The major impact of climatic fluctuations on antioxidant potential and lipid biosynthesis has been documented [65,66]. The exceptionally high F-value for DPPH suggests particular sensitivity of lipophilic antioxidants to environmental conditions [26].

The significant varietal effect demonstrates substantial genetic diversity, although lower F-values than year effects indicate overall stronger environmental influence. The Year × Genotype interaction complicates direct recommendations and underscores the need for multi-environmental evaluations [67].

The PCA results indicate reduced biochemical complexity in 2024, likely due to environmental constraints [68,69].

The trade-off between antioxidant capacity and lipid productivity in 2023, and the decoupling between antioxidant activity and phenolic concentration in 2024, indicate environmental contingency of the metabolic trade-off. This rotation demonstrates that the balance between competing metabolic pathways is not fixed but responds dynamically to environmental conditions.

These results highlight three major findings: (1) biochemical diversity is dynamically modulated by genotype and environment, (2) the negative association between antioxidants and yield confirms allocation constraints [70,71], and (3) identification of stably performing genotypes enables reliable selection for commercial contexts.

The moderate TPC-TFC correlation (r > 0.50) indicates divergent regulation at biosynthetic branch points [52]. The variable contributions of phenolic compounds to antioxidant potential are consistent with findings by Sánchez-Rangel et al. (2013) [59].

The negative correlation between oil content and TPC/TFC underscores the trade-off between secondary metabolites and lipid accumulation under stress [52]. The weak correlation between seed yield and oil content suggests that oil concentration per unit weight varies considerably across genotypes [72].

Overall, these results demonstrate that genetic and environmental variability play a determining role in biochemical quality. The 2024 season, characterized by moderate water stress, favored phenolic accumulation and antioxidant activity, while 2023 promoted lipid synthesis. These divergent responses confirm that genotype × environment interactions influence metabolic partitioning [73].

### 4.4. Interpretation of FTIR-ATR Spectral Features

This spectral similarity reflects the conserved biochemical composition of fig seed oils and seeds, largely dominated by lipids, phenolic derivatives, proteins, and carbohydrates, in agreement with previous reports on fig and other oil-rich seeds [74,75].

These signals reflect the presence of –CH_2_ and terminal –CH_3_ groups from saturated and unsaturated fatty acids, confirming the lipidic nature of fig oils [39,40]. The intensity ratio information on chain length distribution is supported by studies showing higher ratios associated with longer fatty acid chains [76].

This band is diagnostic for oil content and esterification degree, with its position and intensity sensitive to the degree of fatty acid saturation and oxidation state [76].

The relative intensity correlation with phenolic content (r = 0.42, *p* < 0.01) confirms that the fingerprint region contains meaningful information about phenolic composition. The low-frequency band, though often overlooked, contains valuable information about seed matrix composition and can aid in varietal discrimination [77].

These differences are not unexpected, since interannual variations in plant secondary metabolites and oil biosynthesis are strongly dependent on climatic and environmental conditions such as rainfall distribution, irrigation availability, and temperature stress [52,62].

From a biochemical standpoint, the enhanced absorbance in the O–H band in 2024 could reflect a higher accumulation of phenolic hydroxyl groups and water-associated hydrogen bonding, often linked to stress-induced enhancement of antioxidant defenses [43,44]. However, this spectral observation contradicts the biochemical data showing reduced TPC in 2024 (31.2 vs. 48.7 mg GAE/100 g of oil), suggesting that the increased O-H absorbance may instead reflect altered moisture content, increased glycosylation of phenolics, or changes in hydrogen bonding networks rather than absolute phenolic concentration. This discrepancy highlights the importance of integrating spectral and wet-chemical methods for comprehensive quality assessment.

Meanwhile, the more pronounced C=O ester stretching at 1745 cm^−1^ is indicative of increased levels of triacylglycerols and fatty acids, suggesting that lipid biosynthesis was favored under the 2024 climatic conditions, though, again, this contradicts the measured decrease in oil yield from 26.7% to 21.2%. The apparent inconsistency may reflect compositional changes, specifically, an increase in the proportion of esterified lipids relative to free fatty acids or polar lipids rather than total lipid content, possibly due to differences in temperature or water stress at seed filling [41,42]. These observations underscore that FTIR absorbance intensities reflect molecular composition and structural organization rather than simple component concentrations, necessitating multivariate calibration models to extract quantitative information [78].

### 4.5. Integrated Areas as Discriminatory Markers4.5.1. Yearly Differentiation of Integrated Areas

The shift at 3012 cm^−1^ in 2024 is consistent with stress-induced enrichment [79]. Enhanced methylene vibrations at 2928 cm^−1^ indicate structural adjustments. Higher carbonyl peak areas support the hypothesis of increased structural order in lipids [41], with enrichment in saturated or monounsaturated fatty acids [76].

Structural adjustments reflected in the 1375 cm^−1^ band and increased carbohydrate-related absorbance at 1050 cm^−1^ align with biochemical observations showing decreased phenols but increased DPPH reactivity [62,80]. These modifications suggest coordinated metabolic responses to environmental conditions.

#### Varietal Differentiation of Integrated Areas

The abundance of hydroxylated compounds in ‘Palmares’, ‘Ahra’, and ‘Breba Blanca’ has been previously reported (Hssaini et al., 2021) [81]. Greater abundance of long-chain fatty acids in ‘Palmares’, ‘INRA 2307’, and ‘Dottato Perguerolles’ reflects genotypic differences in desaturase enzyme activity [82,83].

Higher esterification efficiency in ‘Dottato Perguerolles’ and ‘Bourjassate Blanca’ is consistent with lipid metabolism differences [42]. The genotypic clustering observed in bands at 1375 and 1050 cm^−1^ has been noted in other studies [84].

This spectral variability underscores genetic control of macromolecular composition, supporting the hypothesis of distinct biochemical strategies underlying varietal adaptation [12,85].

### 4.6. Interpretation of Multivariate Analysis of FTIR-ATR Integrated Areas

These interaction effects indicate that genotypes respond differentially to environmental conditions, with some cultivars maintaining stable spectral profiles across years (e.g., ‘VCR 276/49’, ‘Condria’) while others exhibit pronounced year-dependent shifts (e.g., ‘Ferqouch Jmel’, ‘Ahra’, ‘Filalia’). This genotype-specific environmental plasticity reflects differential regulation of lipid desaturation, phenolic hydroxylation, and esterification pathways under thermal and water stress [76,81].

The PCA results demonstrate that year-dependent metabolic plasticity modulates the biochemical signature. The interannual variability governed by shifts in the balance between carbonyl compounds and carbohydrates reflects environmental adaptation strategies. Clear varietal groupings in 2023 and more compact distribution in 2024 confirm environmental homogenization while maintaining genotypic identity. These results demonstrate that lipid and carbohydrate vibrations serve as robust varietal identity markers, while the carbonyl region is a sensitive indicator of environmental modulation [78,86]. The isolation of ‘Bourjassate Blanca’ indicates an atypical composition worthy of further investigation for unique biochemical properties.

## 5. Conclusions

This study examined biochemical and spectral variability in seed oils from 37 fig genotypes over two harvest seasons (2023–2024) to determine whether genotype × environment interaction drives coordinated metabolic shifts detectable by FTIR-ATR spectroscopy. Results confirmed this hypothesis through multiple lines of evidence. Statistical analysis demonstrated highly significant effects (*p* < 0.001) of year, variety, and their interaction on all measured parameters. Total phenolic content decreased 36% between years (48.7 to 31.2 mg GAE/100 g oil), while DPPH scavenging activity increased 34% (43.7% to 58.5%), indicating a compositional shift toward lipophilic antioxidants under changing environmental conditions. Concurrently, oil yield declined from 26.7% to 21.2%, exhibiting a negative correlation with phenolic levels (r = −0.49, *p* < 0.001), which supports the existence of metabolic competition between lipid biosynthesis and secondary phenolic pathways. Among tested varieties, ‘Dottato Perguerolles’, ‘VCR 276/49’, and ‘Condria’ maintained consistently favorable biochemical profiles across both seasons. FTIR-ATR spectroscopy successfully captured these genotype–environment-driven variations through diagnostic absorption bands at 3012, 2928, 1745, 1375, and 1050 cm^−1^, with variance analysis confirming substantial spectral effects (F > 10^3^, *p* < 0.001). Multivariate analysis explained 75.4–84.5% of total variance and achieved clear discrimination between genotypes and harvest years, validating the approach for rapid cultivar authentication and quality assessment. These findings establish that fig seed oil composition responds predictably to genotype–environment interactions, with FTIR-ATR providing a practical tool for quality monitoring. Future work should incorporate multi-location trials across Mediterranean environments, employ targeted metabolomics and lipidomics to identify specific bioactive compounds, and integrate molecular approaches, including transcriptomics and genome-wide association mapping, to enable marker-assisted breeding for stable, high-value fig seed oil production.

## Figures and Tables

**Table 1:** List of exotic and local genotypes of fig trees (*Ficus carica* L.) used in this study.

Local Genotypes	Exotic Varieties
INRA 2307	White Adriatic
Fassi	VCR 276/49
Ournakssi 2214	Sarilop
Filalia	Palmares
INRA 1308	Bourjassate Noire
Bioudi 2222	Breba Blanca
El Khal	Bourjassate Blanca
Jeblia	Breba Negra
Noukali	Conidria
INRA 1304	Cuello Dama Blanca
Hamra	Rhamam
INRA 2603	Dottato Perguerolles
Bioudi 2878	Sucre Vert
Ahra	Pingo de Mel
INRA 2501	
Beida	
Embar Lebied	
INRA 2304	
Hmidi	
Ferqouch Jmel	
Ournakssi 2282	
INRA 1312	
Tarnimt	

**Table 2:** Numerical coding of thirty-seven fig (*Ficus carica* L.) varieties used in biochemical and FTIR-ATR analyses.

1: Ahra	20: INRA 1308
2: Beida	21: INRA 1312
3: Bioudi 2222	22: INRA 2304
4: Bioudi 2878	23: INRA 2307
5: Bourjassate Blanca	24: INRA 2501
6: Bourjassate Noire	25: INRA 2603
7: Breba Negra	26: Sarilop
8: Breba Blanca	27: Jeblia
9: Conidria	28: Noukali
10: Cuello Dama Blanca	29: Ournakssi 2214
11: Dottato Perguerolles	30: Ournakssi 2282
12: El Khal	31: Palmares
13: Embar Lebied	32: Pingo de Mel
14: Fassi	33: Rhamam
15: Ferqouch Jmel	34: Sucre Vert
16: Filalia	35: Tarnimt
17: Hamra	36: VCR 276/49
18: Hmidi	37: White Adriatic
19: INRA 1304	

**Table 3:** Seeds yield (g/fruit) and oil content (%) of studied thirty-seven genotypes between the 2023 and 2024 harvests. (mean ± standard deviation) values of three repetitions.

Year	Variety	Seeds Yield (g/Fruit)	Oil Yield (%)
2023	INRA 2603	2.239 ± 0.043	31.8 ± 1.06
	Ferqouch Jmel	1.68 ± 0.04	30.84 ± 0.92
	INRA 2304	2.154 ± 0.051	34.41 ± 1.0
	INRA 2307	1.492 ± 0.072	21.77 ± 0.75
	Sarilop	2.701 ± 0.135	32.83 ± 0.82
	Ahra	3.108 ± 0.052	28.51 ± 0.85
	Beida	0.733 ± 0.051	27.25 ± 0.65
	Bioudi 2222	3.133 ± 0.065	28.51 ± 0.87
	Bioudi 2878	3.089 ± 0.021	34.56 ± 0.95
	Bourjassate Blanca	1.166 ± 0.047	16.42 ± 0.95
	Bourjassate Noire	3.608 ± 0.1	29.45 ± 0.78
	Breba Negra	2.169 ± 0.029	12.67 ± 0.83
	Breba Blanca	2.424 ± 0.095	19.61 ± 1.22
	Conidria	1.459 ± 0.038	13.55 ± 0.93
	Cuello Dama Blanca	1.562 ± 0.045	12.67 ± 1.21
	Dottato Perguerolles	1.523 ± 0.073	30.32 ± 0.88
	El Khal	2.062 ± 0.035	24.5 ± 0.88
	Embar Lebied	1.841 ± 0.041	31.53 ± 1.13
	Fassi	1.747 ± 0.078	28.03 ± 0.83
	Filalia	1.89 ± 0.041	21.77 ± 0.89
	Hamra	1.964 ± 0.039	34.43 ± 1.02
	Hmidi	2.848 ± 0.059	29.44 ± 0.77
	INRA 1304	1.468 ± 0.025	32.52 ± 0.87
	INRA 1308	2.152 ± 0.099	30.8 ± 0.95
	INRA 1312	2.599 ± 0.001	29.52 ± 1.12
	INRA 2501	2.24 ± 0.07	27.38 ± 0.92
	Jeblia	1.023 ± 0.019	10.55 ± 0.75
	Noukali	1.559 ± 0.061	27.51 ± 1.06
	Ournakssi 2214	1.553 ± 0.068	26.53 ± 0.78
	Ournakssi 2282	1.875 ± 0.087	27.56 ± 0.81
	Palmares	3.001 ± 0.101	27.44 ± 1.09
	Pingo de Mel	1.47 ± 0.045	26.63 ± 1.05
	Rhamam	2.882 ± 0.04	17.53 ± 1.1
	Sucre Vert	1.771 ± 0.071	35.56 ± 1.16
	Tarnimt	2.137 ± 0.064	27.53 ± 0.95
	VCR 276/49	1.585 ± 0.043	30.62 ± 1.11
	White Adriatic	1.666 ± 0.089	34.53 ± 1.08
2024	INRA 2603	4.16 ± 0.096	21.87 ± 1.44
	Ferqouch Jmel	1.323 ± 0.055	37.82 ± 0.43
	INRA 2304	2.84 ± 0.07	26.53 ± 0.23
	INRA 2307	1.405 ± 0.049	23.97 ± 0.18
	Sarilop	4.293 ± 0.104	9.58 ± 1.26
	Ahra	1.767 ± 0.09	26.09 ± 1.04
	Beida	1.713 ± 0.11	12.06 ± 0.22
	Bioudi 2222	2.527 ± 0.074	25.7 ± 0.27
	Bioudi 2878	2.227 ± 0.07	27.3 ± 0.68
	Bourjassate Blanca	1.643 ± 0.076	10.78 ± 0.27
	Bourjassate Noire	5.053 ± 0.07	25.7 ± 1.1
	Breba Negra	1.373 ± 0.055	24.22 ± 0.35
	Breba Blanca	1.84 ± 0.06	13.75 ± 0.73
	Conidria	1.813 ± 0.111	12.23 ± 0.31
	Cuello Dama Blanca	2.847 ± 0.07	24.1 ± 0.91
	Dottato Perguerolles	0.717 ± 0.06	7.81 ± 0.21
	El Khal	1.96 ± 0.105	21.05 ± 1.43
	Embar Lebied	2.063 ± 0.131	21.55 ± 0.38
	Fassi	1.403 ± 0.071	27.85 ± 1.69
	Filalia	2.213 ± 0.085	27.74 ± 0.19
	Hamra	2.027 ± 0.08	19.0 ± 0.96
	Hmidi	1.68 ± 0.105	19.36 ± 3.0
	INRA 1304	0.387 ± 0.055	6.31 ± 0.69
	INRA 1308	1.64 ± 0.08	17.6 ± 0.25
	INRA 1312	3.855 ± 0.106	16.54 ± 0.79
	INRA 2501	3.06 ± 0.111	30.66 ± 0.32
	Jeblia	1.837 ± 0.08	12.83 ± 0.33
	Noukali	2.957 ± 0.15	30.4 ± 0.36
	Ournakssi 2214	2.38 ± 0.075	24.84 ± 0.75
	Ournakssi 2282	0.653 ± 0.05	28.52 ± 1.1
	Palmes	3.43 ± 0.075	11.98 ± 0.92
	Pingo de Mel	1.827 ± 0.065	16.7 ± 0.44
	Rhamam	1.477 ± 0.08	23.43 ± 0.47
	Sucre vert	2.917 ± 0.075	17.33 ± 0.4
	Tarnimt	0.747 ± 0.06	28.2 ± 0.36
	VCR 276/49	4.867 ± 0.125	24.74 ± 0.88
	White Adriatic	2.217 ± 0.096	30.11 ± 1.32

**Table 4:** Descriptive biochemical and agronomic parameters of fig oils in 2023 and 2024.

	Year	Mean	Std	Min	25%	50%	75%	Max
TPC (mg GAE/100 g of oil)	2023	48.678	16.593	16.522	38.859	45.543	54.13	103.478
TFC (mg QCE/100 g of oil)		15.616	5.856	5.09	12.591	14.545	16.773	37.636
ABTS (%)		32.343	14.283	6.583	21.289	33.615	44.726	56.821
DPPH (%)		43.698	20.944	12.23	30.083	36.676	54.081	86.865
Oil yield (%)		26.683	6.771	9.86	24.015	28.42	31.303	36.74
Seeds yield (g/fruit)		2.041	0.657	0.688	1.547	1.924	2.469	3.702
TPC (mg GAE/100 g of oil)	2024	31.205	16.639	10.761	22.011	27.717	36.304	115.109
TFC (mg QCE/100 g of oil)		11.775	1.883	9.273	10.818	11.455	12.182	19
ABTS (%)		26.313	6.097	16.807	21.989	25.35	29.062	53.221
DPPH (%)		58.487	10.779	34.063	54.54	62.143	66.961	72.306
Oil yield (%)		21.245	7.423	5.52	16.193	22.9	26.688	38.3
Seeds yield (g/fruit)		2.247	1.119	0.33	1.56	1.94	2.84	5.12

**Table 5:** Results of the Analysis of Variance (ANOVA) for the effects of year, variety and their interaction on biochemical and yield traits of fig oils.

Source	Variable	DF	F_Value	*p*_Value
Year (Y)	TPC (mg GAE/100 g of oil)	1	1372.844	0.000
	TFC (mg QCE/100 g of oil)	1	828.364	0.000
	DPPH (%)	1	6199.064	0.000
	ABTS (%)	1	197.374	0.000
	Oil yield (%)	1	1864.592	0.000
	Seeds yield (g/fruit)	1	403.740	0.000
Variety (V)	TPC (mg GAE/100 g of oil)	38	71.164	0.000
	TFC (mg QCE/100 g of oil)	38	62.087	0.000
	DPPH (%)	38	449.107	0.000
	ABTS (%)	38	35.881	0.000
	Oil yield (%)	38	188.746	0.000
	Seeds yield (g/fruit)	38	550.762	0.000
Y:V	TPC (mg GAE/100 g of oil)	38	54.554	0.000
	TFC (mg QCE/100 g of oil)	38	44.925	0.000
	DPPH (%)	38	367.332	0.000
	ABTS (%)	38	28.613	0.000
	Oil yield (%)	38	139.474	0.000
	Seeds yield (g/fruit)	38	282.623	0.000

**Table 7:** Statistical significance of year, variety and their interaction on the main integrated FTIR bands in fig seed oils samples.

Source	Wavenumber (cm^−1^)	DF	F_Value	*p*_Value
Year (Y)	3012 cm^−1^	1	1880.703	0.000
	2928 cm^−1^	1	73,421.83	0.000
	1745 cm^−1^	1	1212.549	0.000
	1375 cm^−1^	1	12,226.9	0.000
	1050 cm^−1^	1	9435.603	0.000
Variety (V)	3012 cm^−1^	38	115.235	0.000
	2928 cm^−1^	38	2268.957	0.000
	1745 cm^−1^	38	24.95263	0.000
	1375 cm^−1^	38	411.1868	0.000
	1050 cm^−1^	38	230.7173	0.000
Y:V	3012 cm^−1^	38	168.4024	0.000
	2928 cm^−1^	38	3082.04	0.000
	1745 cm^−1^	38	24.48441	0.000
	1375 cm^−1^	38	576.5568	0.000
	1050 cm^−1^	38	355.0112	0.000

## Data Availability

Data are available upon request.

## Abbreviations

The following abbreviations are used in this manuscript:

ABTS	2,2′-azino-bis (3-ethylbenzothiazoline-6-sulfonic acid)
Ac	Absorbance of Control
Ae	Absorbance of Sample
ANOVA	Analysis of Variance
ATR	Attenuated Total Reflectance
DF	Degrees of Freedom
DPPH	2,2-diphenyl-1-picrylhydrazyl
FRSA	Free Radical Scavenging Activity
FTIR	Fourier-Transform Infrared
FTIR-ATR	Fourier-Transform Infrared–Attenuated Total Reflectance
G × E	Genotype × Environment interaction
GAE	Gallic Acid Equivalent
HSD	Honest Significant Difference
INRA	National Institute of Agricultural Research
IR	Infrared
MA	Morocco (Maroc)
PC1	Principal Component 1
PC2	Principal Component 2
PCA	Principal Component Analysis
QCE	Quercetin Equivalent
R^2^	Coefficient of Determination
Std	Standard Deviation
TFC	Total Flavonoid Content
TPC	Total Phenolic Content
UK	United Kingdom
USA	United States of America
UV-Vis	Ultraviolet-Visible
V	Variety
Y:V	Year × Variety Interaction
Y	Year
